# Structural correlations in bacterial metabolic networks

**DOI:** 10.1186/1471-2148-11-20

**Published:** 2011-01-20

**Authors:** Sebastian Bernhardsson, Philip Gerlee, Ludvig Lizana

**Affiliations:** 1 Center for Models of Life, Niels Bohr Institute, Blegdamsvej 17 DK-2100 Copenhagen Ø, Denmark; 2 Wallenberg Laboratory, Institute of Medicine, Göteborg University, Göteborg, Sweden

## Abstract

**Background:**

Evolution of metabolism occurs through the acquisition and loss of genes whose products acts as enzymes in metabolic reactions, and from a presumably simple primordial metabolism the organisms living today have evolved complex and highly variable metabolisms. We have studied this phenomenon by comparing the metabolic networks of 134 bacterial species with known phylogenetic relationships, and by studying a neutral model of metabolic network evolution.

**Results:**

We consider the 'union-network' of 134 bacterial metabolisms, and also the union of two smaller subsets of closely related species. Each reaction-node is tagged with the number of organisms it belongs to, which we denote organism degree (OD), a key concept in our study. Network analysis shows that common reactions are found at the centre of the network and that the average OD decreases as we move to the periphery. Nodes of the same OD are also more likely to be connected to each other compared to a random OD relabelling based on their occurrence in the real data. This trend persists up to a distance of around five reactions. A simple growth model of metabolic networks is used to investigate the biochemical constraints put on metabolic-network evolution. Despite this seemingly drastic simplification, a 'union-network' of a collection of unrelated model networks, free of any selective pressure, still exhibit similar structural features as their bacterial counterpart.

**Conclusions:**

The OD distribution quantifies topological properties of the evolutionary history of bacterial metabolic networks, and lends additional support to the importance of horizontal gene transfer during bacterial metabolic evolution where new reactions are attached at the periphery of the network. The neutral model of metabolic network growth can reproduce the main features of real networks, but we observe that the real networks contain a smaller common core, while they are more similar at the periphery of the network. This suggests that natural selection and biochemical correlations can act both to diversify and to narrow down metabolic evolution.

## Background

Evolution of metabolism occurs through the acquisition and loss of genes whose products acts as enzymes in metabolic reactions. From a presumably simple primordial metabolism the organisms living today have evolved complex and highly variable metabolisms, ranging from extremophiles thriving in extreme conditions and feeding on inorganic compounds such as ironsulfide, to endosymbiontic parasites who are dependent on their hosts and cannot themselves synthesise essential compounds like aminoacids. In spite of this diversity there are still reactions and pathways which are found in a large number of organisms, such as the glycolytic pathway, which probably emerged early in the history of life on earth [1]. Changes to the metabolism are constrained by the available genetic variation, the laws of biochemistry and by the selective pressure the organisms are subject to. Among eukaryotes the main source of evolutionary novelty is gene duplication [2,3], whereby unequal cross-over of a section of a chromosome leads to the duplication of the corresponding genes. The loss of selective pressure on one copy can then lead to the divergence and sometimes a new functionality of the duplicated gene. Prokaryotes, on the other hand, make use of horizontal gene transfer (HGT) [4], where genes can be transferred among organisms even from different species. This is a powerful mechanisms which allows for rapid adaptation to new conditions and possibly to swift changes in metabolic capacities. Several models have been proposed for the evolution of metabolism, the most prominent being the retrograde [5] and the patchwork model [6]. The retrograde model states that metabolic pathways evolve from the core and outwards from a key-metabolite assumed to have been abundant in the environment of the ancestral organism. Depletion of the key-metabolite then led to a selective advantage for organisms that could synthesise the missing metabolite from some other metabolite in the environment. This process is thought to have been repeated giving rise to outward-growing pathways. The patchwork model on the other hand assumes a low substrate-specificity to enzymes in the early stages of evolution, so that each enzyme could catalyse several reactions. Gene duplication events followed by divergence in function then lead to the refinement and specialisation among the enzymes which presumably gave rise to the structure found in metabolic networks today. These two models are however not mutually exclusive, and evidence has been found in support of both. For example homologous enzymes containing a specific domain (TIM-barrels) have been found in several distinct pathways [7], supporting the patchwork model, but there is also evidence that homologous enzymes are commonly found within the same pathway [8]. Horizontal gene transfer (HGT), which is prominent among prokaryotes, allows for a different mode of metabolic evolution, where enzymes tend to be transferred in groups which are functionally coupled. This would lead to a growth pattern similar to the retrograde model, but not necessarily with the genes being homologous. Further, it has been shown that the genes encoding for enzymes that are involved in transport reactions and reactions far away from the core of the network are more variable in evolution [9]. That study was however only carried out on *E. coli *and its closest neighbours in the γ-proteobacteria class, and more thorough studies are required to draw more accurate conclusions. In this paper we take a broader view by incorporating 134 bacteria from 16 phyla, and utilising a network perspective to gain quantitative insight into structural correlations between metabolic networks of different species. Comparative studies of microbial metabolic networks have shown that the overlap in metabolic capability between species correlates well with evolutionary kinship, and that phylogenetic trees based on this metric are similar to those which are based on more traditional measures of similarity in 16 S rRNA [10,11]. It has also been shown that typical network measures such as modularity and average path length have a good predictive power when it comes to clustering metabolic networks into phylogenetic groups [12]. The properties of the reaction nodes themselves was shown to correlate with the number of organisms the reactions are found in. In particular the betweenness centrality, which measures the number of shortest paths in the network passing through the node, was a good predictor, while the node degree itself was a poor predictor, i.e. nodes with high connectivity are not typically present in more organisms [13]. Looking at correlations between pairs of reactions Wagner [14] showed that some reaction pairs are likely to occur together in one organism, while others are rarely found together. This suggests that evolution of metabolic networks is governed by reaction combinations that are favoured by natural selection. The study by Wagner, however, does not take into account the biochemical network structure these reactions form, and there are many other questions yet unanswered. How are these correlated reactions interconnected in the network? How are reactions added and removed through the evolution of the metabolism, and to what extent does a common metabolic connected core exist? In the present study we aim at an understanding of metabolic network evolution in the context of the structural constraints imposed by existing biochemistry. By comparing which parts, their topological location, and to what degree the metabolic networks of different bacterial species overlap we hope to gain insight into the evolutionary history which has shaped these networks. A few studies have dealt with related topics such as the extent of which network modularity [15-17] and synthesising capabilities [18] correlates among species in the ancestral tree, but have not done so from a topological perspective. As a further comparison we will also make use of a simple model of network growth, which will serve as a model of a scenario where evolution is completely neutral and only controlled by biochemical constraints. Our analysis presented here indicates that reactions with similar organism degree (OD), denoting the number of organisms in which the reaction is present, tend to be found structurally close to each other in the metabolic network and those with high OD are in general located in proximity of the centre.

## Results

We have focused on the reaction-representation of the metabolic networks of 134 bacterial species. The metabolism of an organism can conveniently be represented by a network, which consists of two types of nodes: reactions and metabolites. In the network a directed link connects a metabolite and a reaction if that metabolite is a substrate of the reaction, and conversely a link connects a reaction node and a metabolite if the metabolite is produced in that reaction. However, for simplicity one often considers a projected network where only reaction nodes are present. This means that the nodes in the network represent metabolic reactions, and there is a directed link between two nodes if any of the products of one reaction is the substrate of the other. We have studied both the metabolic networks of a union of 134 bacterial species, as well as those of a subset of two phyla, Chlamydiae and Proteobacteria. One of the key concepts in this work is the organism degree (OD) of a metabolic reaction which denotes the number of different species it is present in. This is of course a crude approximation and ignores species specific information, which could be used for inferring the evolution of metabolic networks. For example, one could from known phylogenies and metabolic networks infer the gain and loss of reaction along the phylogenetic tree, as was done by Pal and colleagues [9]. Furthermore, Borenstein et al. [19] studied the concept of "seed set" of metabolic networks, defined as the minimal subset of metabolites for a given organism that cannot be synthesised by the other metabolites in the network, from which phylogenetic trees could be reconstructed. Another approach could be to analyse the precise overlap between pairs of species, which coupled with phylogenetic distance, could give insight into evolutionary patterns. In the light of this, discarding the phylogenetic profile of every reaction and replacing it by single number might seem a drastic move, but it allows us to view the data from a topological perspective, something that would have been next to impossible if species identity and phylogenetic information was taken into consideration. Further, it allows for the use of methods from graph theory and complex network analysis, which strengthen our approach. The analysis presented in this paper should thus be viewed as a complement to more traditional bioinformatics approaches to inferring the growth of metabolic networks. In order to make sure that the projection from the list of actual species (i.e. phylogenetic profile) onto a single number, the organism degree, for each reaction in the network, was reasonable and information preserving, we measured how often two neighbouring nodes in the reaction network of the same OD in fact are present in precisely the same species. This turned out to be true in 93% of the cases confirming that the OD is a reasonable level of description, and a meaningful concept to study. The remaining 7% correspond to 85 reaction pairs of which a majority are pairs with OD = 1, which, as our analysis will show, lie at the periphery of the network, and are unlikely to have an impact on the main conclusions of our study.

## Illustrating structural correlations in the metabolism by coarse-graining the reaction network

**Figure 1:**
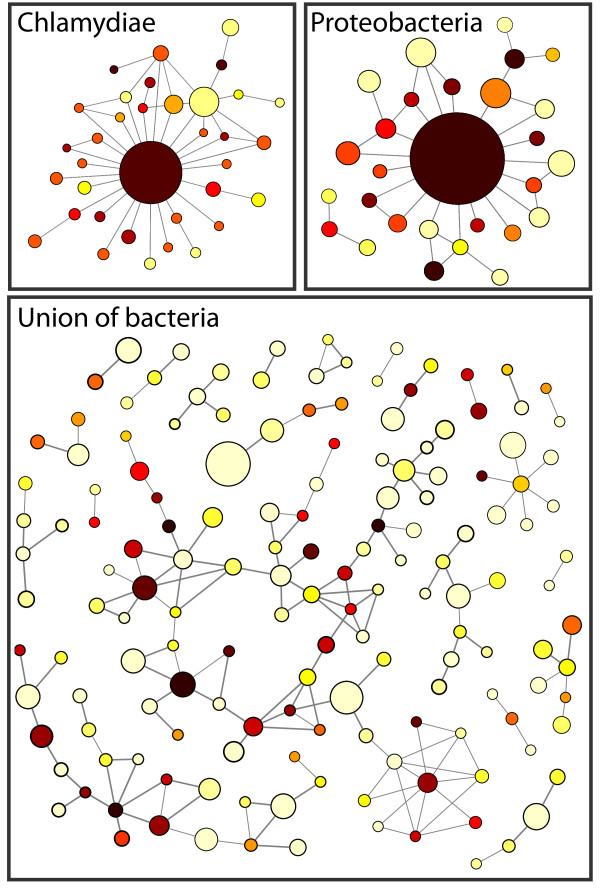
**Coarse-grained metabolic networks for subsets of Chlamydiae, Proteobacteria and the union of bacteria (an exact list of organisms of the respective families is found in additional file**1**,Table S1)**. Neighbouring reaction nodes in the metabolic networks with the same organism degree (OD) are lumped into one super node, the size of which indicates the number of reaction nodes it contains. Dark to light colouring indicate high to low OD level. Two super nodes are connected of there exist at least one link between two reactions in the respective super nodes. See the Methods section for a more detailed description of the coarse-graining procedure.

**Figure 2:**
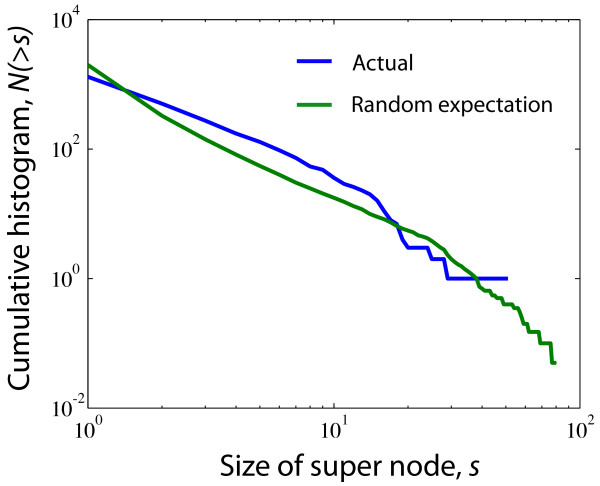
**Histogram of super node sizes for the union of bacteria**. The plot shows that the union-network contains more super nodes of size 1 <*s *< 10, compared to a network where all ODs have been randomised.

In order to visualise structural correlations in the metabolic network graphically we made a coarse-grained representation of the networks where reactions of the same OD were collapsed into one *super node *if there exists a direct link between them (see Methods for more details). The smallest super nodes have been omitted in the figure for the sake of clarity, resulting in a highly disconnected network for the largest union. Figure 1 contains the coarse-grained metabolisms for the union of all 134 bacteria as well as for the union of a subset of species from Chlamydiae and Proteobacteria. The size of each super node reflects the number of reactions it hosts and dark to light colouring indicates high to low OD. From figure 1 we conclude that for the smaller subsets of species, reactions with high OD tend to be connected to each other and found in the centre, while reactions present only in a few organisms tend to be located on the periphery; there is in general a dark to light colour gradient from the most central super node and outward. For the union of all bacteria this trend is not so evident (but can nevertheless be found with quantitative analysis as we show below) suggesting that an ever-present metabolic core for all species does not exist. The histogram (cumulative) of super node sizes for the union of bacteria is shown in figure 2. For comparison, we have also included the case where the OD values of the reactions have been randomly reshuffled (the green curve shows an average of 20 randomisations) thereby effectively removing OD correlations while maintaining the biochemical constraints (i.e. the structure of the reaction network). The reason why the curve for the real data is above the random expectation for *s *> 1 and drops below at *s *= 1 is because there are many more single nodes, i.e. reactions that are not connected to others with the same OD, and fewer large super nodes in the randomised version. This suggests that reactions with the same OD tend to be found close to each other in the real data (at least compared to the case in which the OD is assigned randomly proportional to their occurrence). To quantify the difference between the two distributions in figure 2 we calculated the z-score $Z=\left(\bar{s}-\mu_{\text{rand}}\right)/\sigma_{\text{rand}}$, where $\bar{s}$ is the average super node size of the real distribution, and *μ*_*rand *_and *σ*_*rand *_denote mean and standard deviation of the distribution when the OD is randomised, respectively. The z-score estimates how likely it is that the observed value $\bar{s}$ is drawn from the distribution where the OD is reshuffled. The values $\bar{s}=2.15$*μ*_*rand *_= 1.45 and *σ*_*rand *_= 0.01 give *Z *≈ 70 which corresponds to a p-value of < 2 × 10^-16 ^(i.e the probability to draw *Z *> 70 from a normal distribution is < 2 × 10^-16^). In summary, based on the course grained picture of the metabolic networks we infer that (*i*) metabolic reactions with high OD tend to be located close to the core and (*ii*) that reactions with the same OD are likely to be found close to each other. These conjectures are investigated in more detail below.

## Quantifying structural correlations

**Figure 3:**
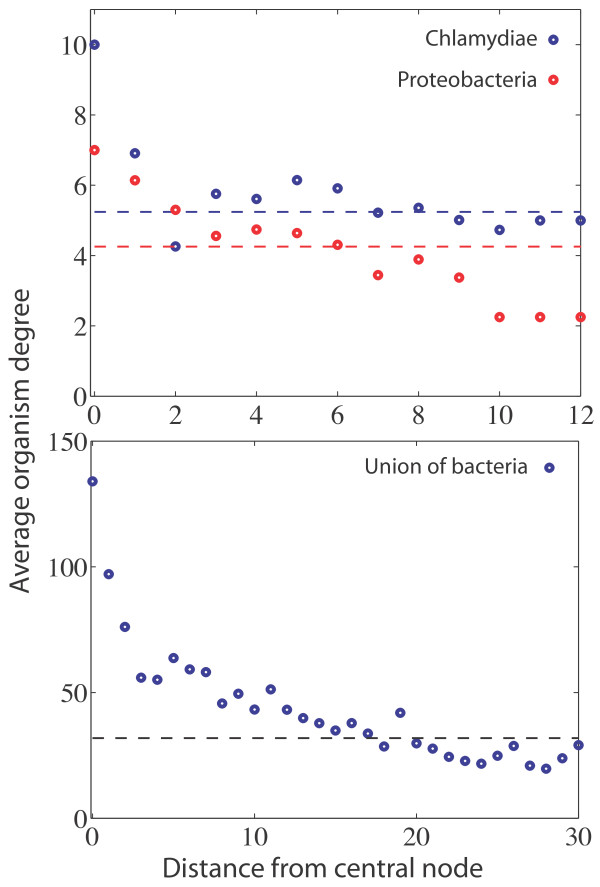
**Average organism degree (OD) as function of the distance from the most central reaction node**. The centre node is defined as the reaction node with the highest betweenness centrality conditioned that it is present in all organisms. The horizontal line is the average OD of all reaction nodes in the network. Both on the family level (Chlamydiae and Proteobacteria) and for the union of bacteria there is a decay in OD when moving outward from the core. This indicates that reactions with high OD are in general found in the centre while species specific reactions with low OD are found further out.

**Figure 4:**
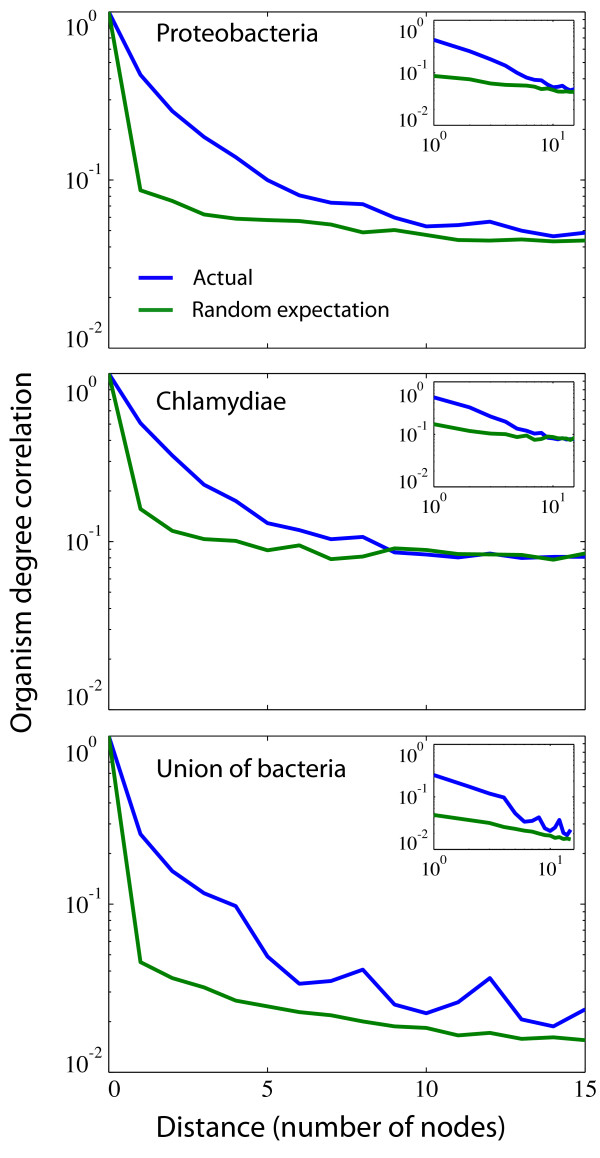
**Structural correlations among reaction nodes with the same organism degree (OD)**. The curves show the two-node correlation function which captures the likelihood of finding two reactions of the same OD *k *steps apart from each other. The blue curve is based on the actual data while the green curve shows the correlation function when the OD attribute of all reaction nodes is randomly reshuffled.

In order to quantify to what extent reaction nodes with high OD are enriched close to the centre of the metabolism we calculated the average OD as a function of distance (number of nodes) from the most central reaction node in the undirected reaction network. The most central reaction was chosen as the one with the highest betweenness centrality conditioned that it was present in all organisms. For the smaller families the reaction with highest betweenness centrality coincided with the maximum OD (in accordance with the study of Liu et al. [13]), while the one for the full bacterial network had OD = 92. The betweenness centrality of a node measures the number of shortest paths that pass through the node, and gives an approximation of the mass transfer through the reaction. Figure 3 shows the average OD of the reactions found at one, two, three etc. steps away from the centre. The trend that reaction nodes with high OD are enriched close to the metabolic core is clear in all three cases but most obvious for the union of bacteria, which shows a steady decline in average OD in the entire measured range. The size distribution of the coarse-grained metabolic network (figure 1) suggests that reactions with the same OD are structurally correlated in the sense that they seem to be found close to each other in the reaction network. In order to quantify this feature further we calculated the likelihood of finding two reactions with the same OD a given number of steps away from each other. This two-node correlation function, averaged over all nodes in the network, is shown in figure 4, and illustrates that there is a clear correlation up to a distance of 5 steps. The correlation is destroyed if the OD values of the reaction nodes are reshuffled which is illustrated by the green curves.

## Model of metabolism governed by biochemistry

**Figure 5:**
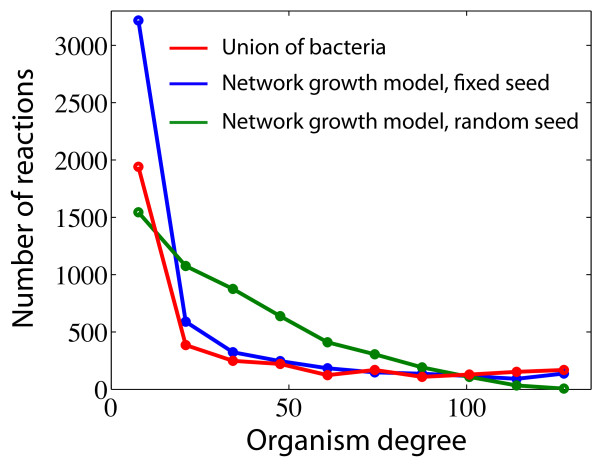
**The distribution of OD in the 'union-network' of bacteria and the two different versions of the model**.

**Figure 6:**
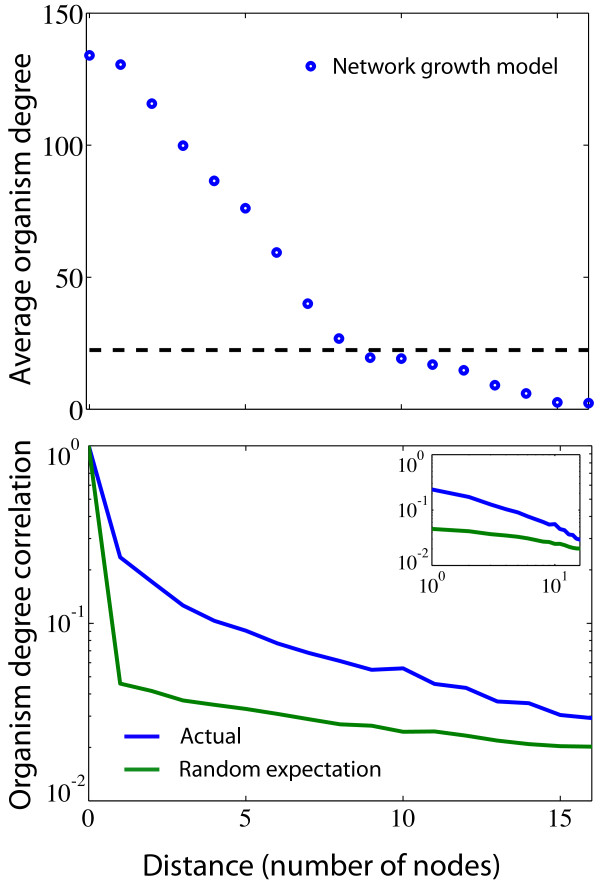
**Analysis of the network growth model analogous to the ones in Figures 3 and 4**. (Top panel) Decay in organism degree (OD) as a function of distance from the most central node. (Bottom panel) The likelihood of finding two reaction nodes of the same OD *k *steps apart.

In order to get an understanding of the interplay between evolution and biochemical constraints on the growth and development of metabolisms we made use of a network growth model which is completely neutral. By this we mean that reactions are removed or added to the metabolism based only on their biochemical compatibility, as defined by the BioCyc database, with the existing metabolism, and no consideration is taken as to the usefulness or change in fitness the addition or removal gives rise to. Starting from a single reaction, we incrementally add reactions which can be linked directly to at least one of the reactions already present in the metabolism (more details are given in the Methods section). The initial, or seed reaction, can be chosen in several ways and we explored two different variants: (i) the initial seed is fixed and chosen as a reaction which is present in all organisms, or (ii) the initial seed is a different random reaction for each generated network. Adding and removing reactions or metabolites based on known metabolisms to construct synthetic metabolic networks is not a new concept. The model presented here is inspired by the work of Maslov et al. [20], where reactions are added (removed) to (from) a given (directed) metabolic network given that the reactions are connected. This means that we in principle could add a reaction for which its ingoing metabolites are not synthesised by our metabolism, and in a more detailed model one should make sure that this condition is satisfied, see e.g. [21,22]. However, it has be demonstrated [23] that when omitting currency metabolites (e.g. water, protons, ATP/ADP, NADH) metabolic pathways are close to linear chains of reactions in which substrates are enzymatically modified in each reaction step. This implies that a considerable fraction of the reactions only have a single substrate (depending of course on the level of coarse graining of currency metabolites) and the mean in and out degree is only slightly above one. We found that the networks produced by our algorithm in the two cases of fixed and random seed reactions are substantially different, where the one with the fixed starting point reproduces features of the real data in a much better way, such as the OD distribution of the reactions making up the metabolism, shown in figure 5. Although the fixed seed scheme over-estimates the number of nodes with low OD, it reproduces the general shape of the curve much better than the random scheme, which exhibits linear rather than an exponential decay in the number of nodes with a given OD. We also grew metabolic networks using an OD = 1 reaction as seed node, and found that the resulting networks were similar, with respect to the measures we are interested in, to the networks generated from the most central node in the complete network (see additional file 1, figure S2). The main reason being that the most central node is included only after a few steps in the algorithm, thus giving rise to similar growth trajectories. Using the fixed seed model, we produced a set of 134 metabolic networks, which were analysed in the same way as the real data (Figures 3 and 4). Figure 6 shows (top panel) the decay in OD when moving outward from the centre, and (bottom panel) to what extent reaction nodes with the same OD are found close to each other compared with the case of when the OD is reshuffled. The average OD of the model networks close to the centre of the network is higher than the real data, but for distances larger than approximately 10 steps from the centre it is considerably lower. Correlations between nodes of the same OD are also prominent in the networks generated with the model, and decay at a rate similar to those observed in the union of bacteria network.

## Is there evidence of an omnipresent metabolic core?

To put numbers on the question of the existence of a commonly shared metabolic core, we analysed the coarse-grained network (figure 1) and calculated core sizes (see Table 1) for the small subset of Chlamydiae and Proteobacteria, the union of bacteria, and for the union of 134 networks generated by our model. We also analysed smaller subsets of randomly assigned families containing 10 organisms, in order to assess the impact of the relatedness of the species and the size of the subsets considered. Two measures were used. First, the size of the largest super node with maximum OD (*s*_core_) compared to the total number of reactions (*N*_tot_) in the network. This measure reflects the size of the metabolic core when compared to the size of the entire network. Second, the size of the largest super node with maximum OD relative to the total number of reactions with maximum OD $\left(N_{{\text{OD}}_{\max}}\right)$ in the coarse-grained network. This measure, on the other hand, quantifies the degree to which the nodes with maximum OD are clustered together in the network. The numbers in the table support what is seen in the figure 1. On the phylum level where kinship between species in general is close, there is a clear connected metabolic core which constitute a significant fraction of the union of their metabolisms. For example, 22% of all metabolic reactions in Chlamydiae from a connected metabolic core, at least among the species in the phylum included here. This should be compared with the average properties of a random subset of ten species, which exhibits a small metabolic core of approximately 1% of the network. However, when merging metabolisms of additional phyla, the core shrinks. For the union of the 134 bacteria studied here, including organisms from 16 phyla, there is only a small fraction of the reactions that can be classified as a shared metabolic core. Analysing the networks generated by our growth model, where metabolic reactions are added one by one starting from a single reaction node without any evolutionary pressure, we found that they show a very well defined metabolic core. However, they are still not dominating the entire metabolism as seen from the small values in the first column. As a robustness test to make up for possible errors due to incomplete annotations in some of the included species, we altered the range of OD values (ΔOD) that allow reactions to be put in the same super node. This also compensates for the natural increase of sparseness in OD with increasing number of organisms. Changing ΔOD effectively means that the size of the measured core also change. However, even though we increase ΔOD from 10 to 50 (which means that all reactions with an OD larger than 100 is included in the core) we still do not get one dominating connected core (Table 1) which means that the above conclusions are insensitive to the choice of coarsening.

**Table 1:** Table of core sizes, where *s*_core _is the size of the largest super node of maximum OD, *N*_tot _is the total number of reactions in the respective reaction network, and $\left(N_{{\text{OD}}_{\max}}\right)$ is the total number of reactions with maximum OD.

Networks	*s*_core_/*N*_tot_	$s_{core}/N_{OD\max}$
Chlamydiae	0.22	0.86
Proteobacteria	0.13	0.70
Random subset of species	0.01	0.21
Union of bact.(ΔOD = 10)	0.004	0.27
Union of bact.(ΔOD = 50)	0.008	0.09
Model (ΔOD = 10)	0.021	~1
Model (ΔOD = 50)	0.079	~1
Random subset of model networks	0.05	~1

The random subsets represents the average result for the union of 10 randomly picked networks from the full set of real organisms and model networks respectively.

## Discussion

The structural correlations between metabolic networks from different species bear a clear mark of their evolutionary history. Species which are closely related usually have more similar metabolisms compared to distant relatives. This feature can clearly be seen in figure 1, which shows the condensed metabolic network of both the union of a wide range of species, and that of phylogenetically closer species. The overlap of the networks restricted to a single phyla are considerably larger, implying a higher similarity in metabolic capability. This picture also suggests that what is common to the species within a phyla forms a connected component, and that the species specific reactions are attached onto this core metabolism. Although this cannot visually be seen for the union of the 134 bacterial species, the quantification of average OD as function of distance from the centre of the network shows that reactions become more specific as we move out to the periphery. The average OD for the two smaller networks decay more rapidly, but this does not imply that the distribution of OD across the network is less structured. On the other hand, as can be seen in figure 4 correlations are prominent. The drop in average OD should instead be interpreted as a consequence of the similarity of the underlying species. A similarity which persists even at the periphery of the network. The two-node correlation function shows that correlations in OD are present up to distance of approximately 5 nodes. This suggests that there is a characteristic length scale within the union-network which tends to be conserved among closely related species. In other words, viewing metabolic evolution as a transfer of subsets of the metabolic network from one species to another (as in HGT), our results suggest that the typical size of these transferred sets of reactions is approximately five. Interestingly, this is close to the average pathway length of 4.4, as defined in the MetaCyc database, suggesting that the annotated pathway structure reflects the structural correlations we have reported on. There is also evidence that pathways are enriched within super nodes (see additional file 1, figure S3), which further supports this view. The importance of HGT in shaping metabolic evolution has previously been highlighted, for example in a study by Pal et. al [9]. They showed that HT-genes are integrated at the periphery of the metabolic network, and that the proportion of HT-genes increases as one moves from biomass producing reactions to nodes at the periphery where transport reactions occur. Further, they showed that pairs of enzymes which were flux coupled were more often gained and lost together in the phylogenetic tree. This concords well with the results presented in this study, that the variability of network structure is higher closer to the periphery, and by taking into account the precise placement of enzymes/reactions on the metabolic union-network we have put forward further quantitative evidence for this growth mechanism. It has been claimed that many of the properties exhibited by metabolic networks such as modularity and scale-free degree distributions are products of adaptive evolution. One argument that has been put forward is for example the robustness and fault-tolerance conferred by a scale-free architecture [24]. However, little emphasis has been put on the underlying biochemical constraints these networks are subject to, and the existing biochemistry has an obvious influence on possible network structures. It has for example been shown that atmospheric reaction networks exhibit scale-free degree distributions [25], and these networks have clearly not been subject to any selective process. On the theoretical side it has also been shown that the scale-free degree distributions of metabolic networks can be obtained as a maximum-entropy solution, giving a random null-model neutral to any selective pressure [26,27]. Naturally the external environment of an organism will have an influence on metabolic evolution, such as in the case of certain endosymbionts which have lost the capability to synthesise amino acids provided by the host, but it is still unclear how much the laws of chemistry act to constrain metabolic evolution. The present study will not resolve these questions, but still gives an idea of how the metabolism of different bacterial species are related with respect to each other. Approximately 1.5% of the nodes in the union-network of all bacteria are present in all species, but these reactions do not form a coherent core, but are distributed across the network. The presence of these universal reactions does suggest some level of constraint, but the fact that they are bridged by different reaction paths in different species on the other hand hints at a degree of plasticity. A large metabolic core was indeed found in the union-networks of the subset of the Chlamydiae and Proteobacteria phyla, but this is an effect of the close phylogenetic relationship of these species, and not of a constraining chemistry. The results of the network growth model also support this dual view. Although the largest super node with maximal OD in the super-network generated from 134 model networks contains almost all the nodes of that OD, it only spans a small fraction of the network. This means that the completely neutral evolutionary process occurring in the model results in metabolisms which exhibit too much overlap compared to the real data, but at the same time are more diverse than the real metabolic networks. Notably the model overestimates the average OD close to the core, while underestimating it further away, an effect which is also obvious from the OD distribution plot (figure 5). This is most likely due to two separate deficiencies of the model. Firstly, nodes with a high degree in the BioCyc-network are more likely to be included in the model networks. The average node degree (in- plus out-degree) in the union-network generated from the model was 4.4, compared to 3.3 for the real data, suggesting that this is the case. These hubs are typically close to the centre of the network, and their affinity to be included in the networks therefore leads to an increased average OD at small distances from the centre. On the other hand, further away from the centre the model gives rise to lower average OD than the real data. This means that the model networks exhibit a larger diversity on the periphery, compared to the bacterial networks which are less diverse at this distance. This discrepancy from the neutral model suggests that natural selection and biochemical correlations can act both to diversify and to narrow down metabolic evolution. Bacterial species sharing the same environment would of course be likely to share pathways which allow for the degradation of compounds specific to that environment [28], and this effect would lead to a lower diversity at the periphery of the metabolic networks. The complimentary roles of some reactions, which were reported as anti-correlated reaction pairs by Wagner [14], could on the other hand lead to a smaller amount of overlap, and thus a smaller number of nodes with high OD. One might argue that the model made use of here is too simple to accurately describe the evolutionary paths of metabolic networks. For example, the model does not take into account the possibilities that the network can shrink (*p *< 1), add/remove chunks of reactions, or that all substrates must be available before a new reaction can be incorporated. This is all very true, however the model is not meant to give a detailed description of the evolutionary path of metabolic networks. Its purpose is to capture how the diversity of possible networks is influenced by the underlying biochemical constraints. In this setting it effectively means that it is the fluctuations of the "surface" of the networks (reactions not included in the common core at the periphery of the network) that is the interesting property, which is independent of the details of the growth model. The model used here is then just the simplest, most straight forward, way of obtaining an ensemble of networks of a given size, grown from a given seed reaction. The growth model has an obvious flavour of the retrograde model of metabolic evolution, where new reactions are added at the periphery of the network. It is therefore not surprising to find that nodes with high OD are found close to the centre of the network and that the average OD decreases as we move out from the centre, but the similar results for the real metabolic networks suggest that an analogous growth process has shaped their evolution. Our results thus point to the retrograde model as being more likely, however this should be looked upon in the light of HGT, as has been suggested in [19].

## Conclusion

We have presented a study of the structural correlations in the metabolic networks of 134 bacterial species. By considering the union-network formed by these networks we were able to show that the universal reactions lie at the centre of the network and more specific reactions are placed closer to the periphery. The analysis of two smaller groups of species taken from the Chlamydiae and Proteobacteria phyla showed that these union-networks exhibited metabolic cores onto which species-specific subnetworks were attached. These features could be reproduced by a simple network growth model confirming that horizontal gene transfer is a prominent mechanism in the evolution of metabolic networks. The model does however overestimate the number of species specific reactions, and also overestimates the size of the common metabolic core, highlighting the effect of environmental selection and biochemical correlations.

## Methods

### Coarse-graining of network

The coarse-graining of the union of the metabolic networks was done by clustering all nodes that are connected to each other, and have the same OD, into one super node, using a depth-first algorithm. The size, *s_i_*, of the super node, *i*, equals the number of reactions that were condensed together. For the small subsets of the phyla Chlamydiae and Proteobacteria two neighbouring reaction nodes are placed in the same super node if their OD is exactly equal. For the much larger set of 134 organisms the sparseness in OD naturally increases, which we compensate by letting each super node represent a range of 10 OD values (e.g. OD_1 _= 1, ..., 10, OD_2 _= 11, ..., 20, etc.). Two super nodes are then joined if there exists at least a reaction in each super node which are linked together. In figure 1 we have omitted the smallest super nodes for the sake of clarity, which explains why the union of all bacteria seems highly disconnected.

### Species under consideration

In our analysis we have done a small-scale analysis on a subset of two bacterial phyla (Proteobacteria and Chlamydiae) as well as a large-scale analysis based on a union of bacterial species taken from the MetaCyc database [29]. In order to have well-defined phylogenetic relationships between the species under consideration we picked 134 species for which a phylogenetic tree has been constructed [30]. Please note that this presents a possible bias where certain phyla are over-represented in terms of DNA-sequencing. The subset of the species from the Proteobacteria were chosen as the 10 closest relatives to *Bordetella parapertusis *(including itself), and the subset of the Chlamydiae was chosen as all the species of that phyla which were included in the phylogenetic study, which amounted to 7 species. A full list and the two subsets of species are given in additional file 1, Table S1.

### Data analysis

The data describing the metabolism of the 134 species was downloaded from MetaCyc (22/10/2009) [29]. We transformed the data into a bipartite network, which contains two types of nodes: metabolites and reactions with directed links between them. Each reaction node was also tagged with a list of species in which it occurs, the organism degree. In order to simplify the analysis, the network was projected to a reaction-reaction network, where two reactions are connected if the product of one is the substrate of the other. However, in order to avoid connections contributed by currency metabolites such as water and ATP, which tend to dominate the network, we applied a pruning algorithm [23], which removed all metabolites with a connectivity higher than 10 prior to the projection. The resulting metabolic network contained 3650 reaction nodes and 4411 links. In order to make sure that the pruning level chosen is appropriate we also generated networks with pruning level 20 and 5. The higher pruning level leads to a dense network with short path lengths, while the lower one gives similar results to pruning level 10. The dependence of average OD as a function of distance on pruning level is shown in additional file 1, figure S1.

### Network growth model

The network growth model used in this study was inspired by a previously published model by Maslov et. al [20], which was used for modelling the evolution of prokaryote metabolism. In our model, the growth of the metabolic network occurs on a background of possible reactions. These were defined from the BioCyc database [31], which describes the union of all known biochemical reactions. This data was downloaded from the BioCyc website (22/10/2009) and subject to the same processing as the MetaCyc-data. In addition we only kept the reactions which were in the giant connected component of the network. This resulted in a network consisting of 5191 reaction nodes and 10130 links. The growth model was implemented as follows: first we define an initial, or seed set of reactions *R*_0_. In each time step *t *of the model we do one of the following: (i) with probability *p *we add a reaction node to *R*_*t*-1 _by at random picking a node which is not in *R*_*t*-1_, but is neighbour of at least one of the current nodes (as defined in the underlying BioCyc-network). With complementary probability 1- *p *we pick a node in *R*_*t*-1_, which is a leaf, i.e. has only one link, and remove it. The following is repeated either until the network defined by *R_t _*reaches a given size or after a given number of time steps. In our version of the model the seed set is only one reaction; either the *Methenyltetrahydrofolate cyclohydrolase*-*reaction *(found in all 134 species) or a randomly chosen reaction, and the parameter *p *was set to unity, meaning that we only add reactions to the network. If we would have used *p *< 1 the end result would have been roughly the same (differing mainly in the collection of peripheral reactions) but would have required more iteration steps. Since we wished to produce a large collection of metabolisms, and not study their exact evolution, we used *p *= 1. The model was run 134 times to produce an ensemble of organisms having the same size as the union of bacteria. Also, the size of each individual network was set to match exactly that of each considered species. In order to make sure that the choice of seed set, in the fixed reaction scheme, does not bias the dynamics of the model we also ran the model with a starting node with OD = 1. The result of this simulation can be found in additional file 1, figure S2, and shows that the union-network in this case exhibits a similar structure.

## Authors' contributions

SB conceived the idea behind the paper, analysed the super-network and co-wrote to the manuscript. PG analysed the MetaCyc-data, implemented the network growth model and co-wrote the manuscript. LL analysed the MetaCyc and model data, and co-wrote the manuscript. All authors have read and approved the final manuscript.

## Supplementary Material

Additional file 1 **Additional table and figures**. The file contains additional Table S1 and additional figures S1-S3. Click here for file
