# Supplementary material for: Structural correlations in bacterial metabolic networks
Source: BMC Evol Biol. 2011 Jan 20;11:20. doi: 10.1186/1471-2148-11-20 (PMC3033826; doi:10.1186/1471-2148-11-20)
Supplement: Additional file 1 — Additional table and figures. The file contains additional Table S1 and additional figures S1-S3. [file 1471-2148-11-20-S1.PDF]

Additional file 1 for “Structural correlations in bacterial metabolic networks” by S. Bernhardsson, P. Gerlee & L. Lizana

Table S1

The species marked with * belong to the Proteobacteria subset and those marked with † belong to the Chalmydiae subset.

Bordetella bronchiseptica RB50*
Bordetella parapertussis 12822*
Bordetella pertussis Tohama I*
Pseudomonas putida F1*
Ralstonia solanacearum GMI1000*
Neisseria meningitidis MC58*
Pseudomonas aeruginosa UCBPP-PA14*
Nitrosomonas europaea ATCC 19718*
Chromobacterium violaceum ATCC 12472*
Chlamydophila pneumoniae AR39†
Chlamydophila pneumoniae CWL029†
Chlamydophila pneumoniae TW-183†
Chlamydophila pneumoniae J138†
Chlamydophila caviae GPIC†
Chlamydia muridarum Nigg†
Chlamydia trachomatis A/HAR-13 †
Streptomyces coelicolor A3(2)
Onion yellows phytoplasma
Nostoc sp. PCC 7120
Synechocystis sp. PCC 6803
Pirellula sp.
Bacillus subtilis subtilis 168
Prochlorococcus marinus AS9601
Staphylococcus aureus aureus Mu50
Staphylococcus aureus aureus N315
Streptococcus pyogenes M1 GAS
Xylella fastidiosa 9a5c
Listeria monocytogenes EGD-e
Streptococcus pneumoniae R6
Staphylococcus epidermidis RP62A
Agrobacterium tumefaciens C58
Xylella fastidiosa Temecula1
Streptococcus pyogenes MGAS8232
Yersinia pestis KIM
Leptospira interrogans serovar Lai str. 56601
Fusobacterium nucleatum nucleatum ATCC 25586
Xanthomonas campestris pv. campestris str. ATCC 33913
Xanthomonas axonopodis pv. citri str. 306
Streptococcus pyogenes SSI-1
Chlorobium tepidum TLS
Clostridium perfringens 13
Corynebacterium efficiens YS-314
Vibrio vulnificus YJ016
Staphylococcus aureus aureus MW2
Corynebacterium glutamicum ATCC 13032

Shigella flexneri 2a str. 301
Streptococcus pyogenes MGAS315
Buchnera aphidicola Sg (Schizaphis graminum)
Tropheryma whipplei Twist
Candidatus Blochmannia floridanus
Streptococcus agalactiae A909
Bifidobacterium longum NCC2705
Salmonella enterica enterica serovar Typhi str. Ty2
Streptococcus mutans UA159
Streptococcus agalactiae NEM316
Shewanella oneidensis MR-1
Clostridium tetani E88
Yersinia pestis CO92
Vibrio vulnificus CMCP6
Tropheryma whipplei TW08/27
Lactobacillus plantarum WCFS1
Oceanobacillus iheyensis HTE831
Pseudomonas syringae pv. tomato str. DC3000
Vibrio parahaemolyticus RIMD 2210633
Aquifex aeolicus VF5
Borrelia burgdorferi B31
Bradyrhizobium japonicum USDA 110
Brucella melitensis 16M
Buchnera aphidicola Bp (Baizongia pistaciae)
Bacteroides thetaiotaomicron VPI-5482
Bacillus cereus ATCC 14579
Coxiella burnetii RSA 493
Streptomyces avermitilis MA-4680
Yersinia pestis biovar Microtus str. 91001
Haemophilus ducreyi 35000HP
Mycobacterium bovis AF2122/97
Helicobacter hepaticus ATCC 51449
Porphyromonas gingivalis W83
Dehalococcoides ethenogenes 195
Deinococcus radiodurans R1
Geobacter sulfurreducens PCA
Mycobacterium avium 104
Photorhabdus luminescens laumondii TTO1
Mycoplasma genitalium G37
Thermotoga maritima MSB8
Treponema denticola ATCC 35405
Treponema pallidum pallidum Nichols
Gloeobacter violaceus PCC 7421
Corynebacterium diphtheriae NCTC 13129
Lactobacillus johnsonii NCC 533
Rhodopseudomonas palustris CGA009
Bacillus anthracis Ames Ancestor
Thermus thermophilus HB27
Bdellovibrio bacteriovorus HD100
Listeria monocytogenes 4b F2365
Sinorhizobium meliloti 1021
Mesorhizobium loti MAFF303099
Mycoplasma mobile 163K
Synechococcus elongatus PCC 6301

Bacillus halodurans C-125
Clostridium acetobutylicum ATCC 824
Lactococcus lactis lactis Il1403
Listeria innocua Clip11262
Mycobacterium leprae TN
Mycoplasma mycoides mycoides SC str. PG1
Mycoplasma penetrans HF-2
Mycoplasma pneumoniae M129
Mycoplasma pulmonis UAB CTIP
Neisseria meningitidis FAM18
Rickettsia conorii Malish 7
Rickettsia prowazekii Madrid E
Thermoanaerobacter tengcongensis MB4
Wolinella succinogenes DSM 1740
Haemophilus influenzae 86-028NP
Photobacterium profundum SS9
Mycobacterium tuberculosis F11
Helicobacter pylori HPAG1
Brucella melitensis biovar Abortus 2308
Buchnera aphidicola Cc (Cinara cedri)
Shigella flexneri 5 str. 8401
Vibrio cholerae O1 biovar El Tor str. N16961
Mycobacterium tuberculosis H37Ra
Streptococcus pneumoniae Hungary19A-6
Ureaplasma parvum serovar 3 str. ATCC 27815
Escherichia coli K-12 substr. MG1655
Fibrobacter succinogenes succinogenes S85
Prochlorococcus marinus MIT 9312
Prochlorococcus marinus MIT 9313
Escherichia coli O157:H7
Synechococcus sp. WH 8102
Helicobacter pylori J99
Desulfovibrio vulgaris Hildenborough
Wolbachia pipientis
Salmonella typhimurium LT2

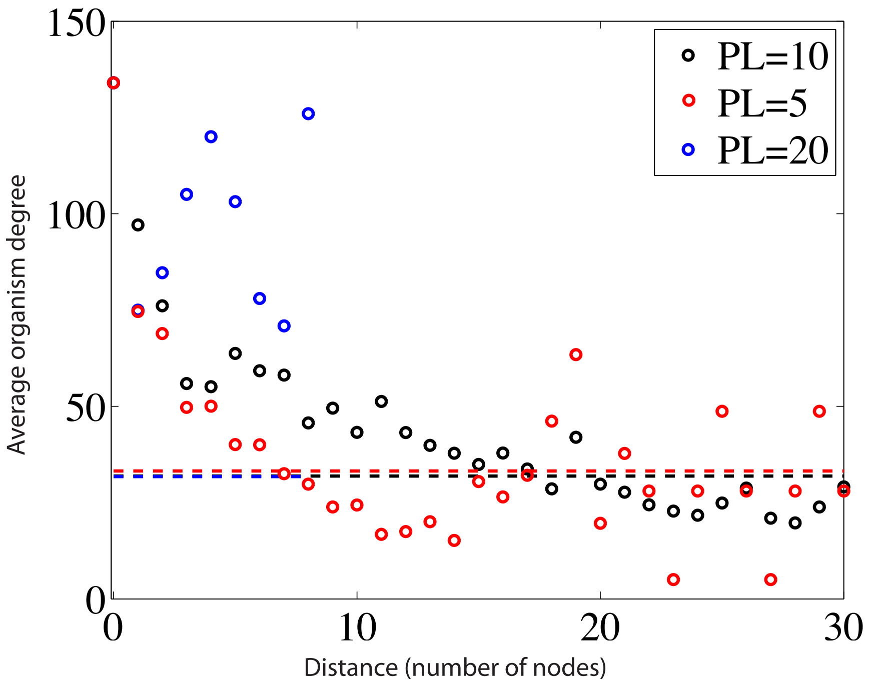

Figure S 1: The dependence of the average OD curve on the pruning level. Pruning level 20 leads to a dense network with short path lengths where the average OD does not seem to correlate with distacne, while the lower one gives similar results to pruning level 10, albeit with a slightly faster decay in average OD.

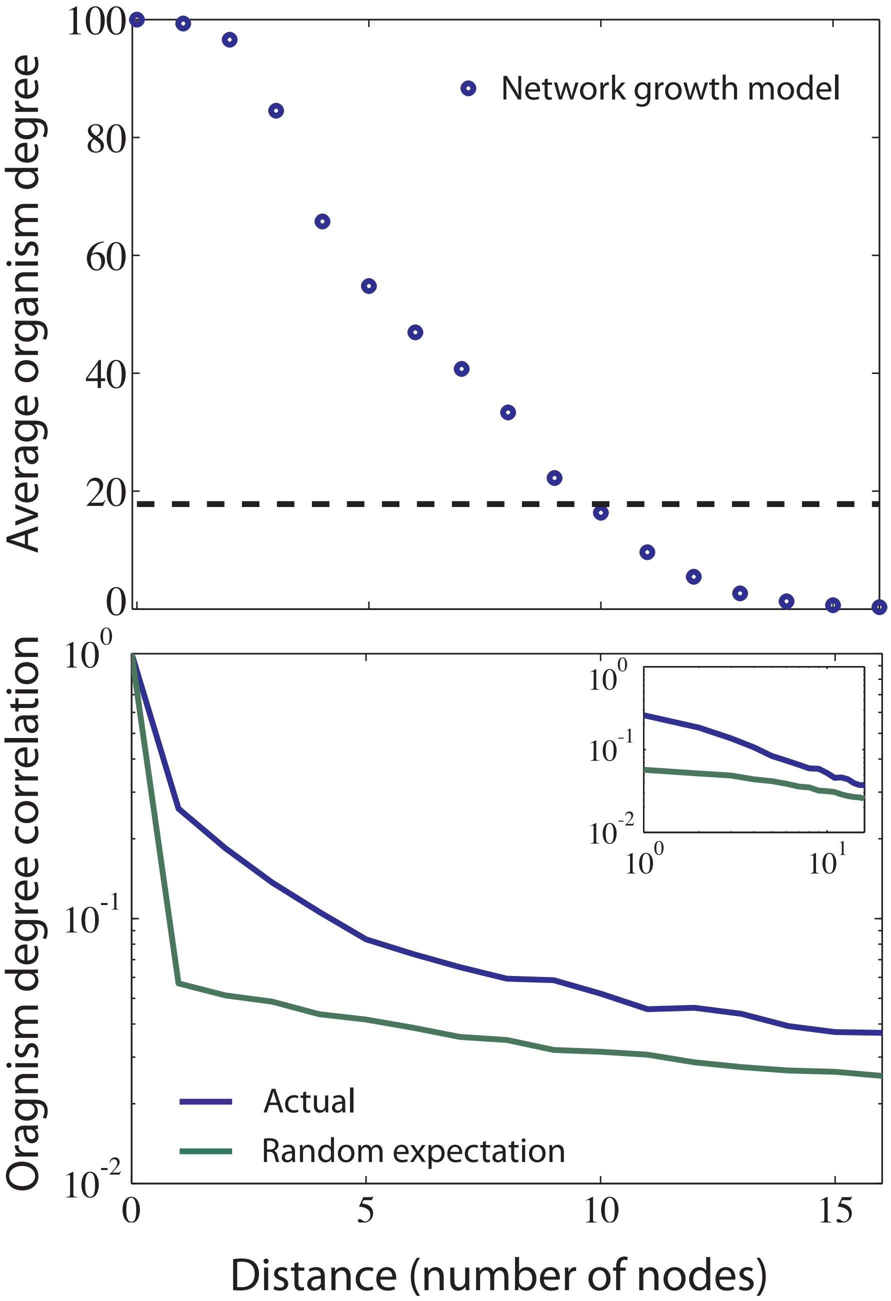

Figure S 2: The properties of the growth model super-network when the starting node was chosen as one with OD=1. The top panel shows the average OD as a function of the distance from the most central node in the network, and lower panel displays the two-node correlation function. The results are similar to those when the starting node was chosen as a node with existed in all 134 bacterial species, and suggests that the model is not sensitive to this choice.

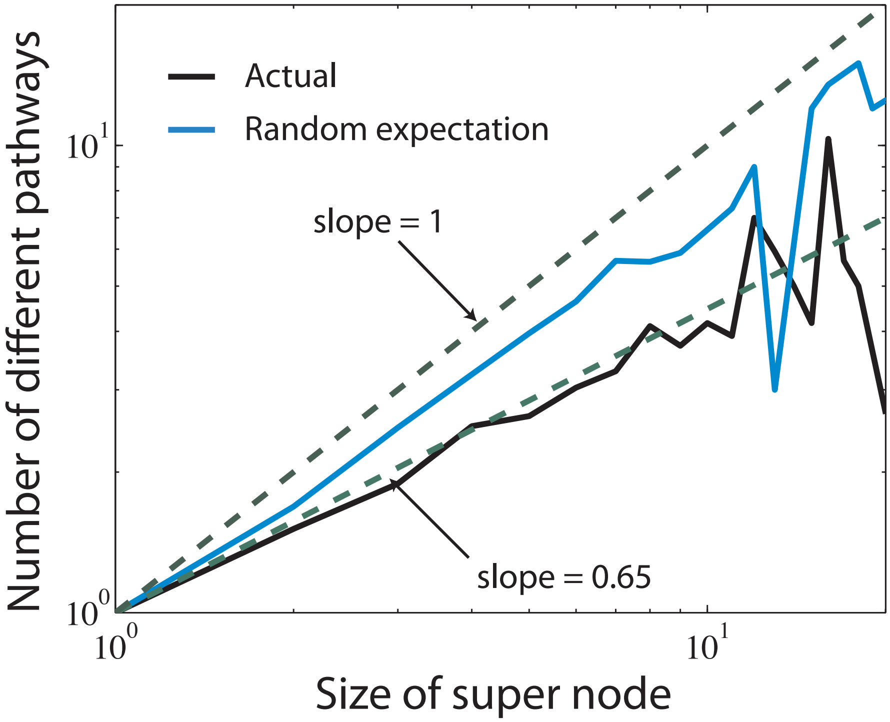

Figure S 3: Pathway enrichment in super nodes. The figure shows the number of unique pathways the super nodes contain as a function of the super node size. If each node within the super node belonged to a distinct pathway the curve would follow the upper dashed with slope one. The real curve has a slope of 0.65 suggesting an enrichment of pathways in the super nodes.
